# Habitual physical activity and sedentary behavior as predictors of dynapenia in older adults: a cross-sectional study

**DOI:** 10.1590/1516-3180.2023.0070.R1.190523

**Published:** 2023-08-25

**Authors:** Lucas dos Santos, Cláudio Bispo de Almeida, Paulo da Fonseca Valença, Rizia Rocha Silva, Isaac Costa Santos, Cezar Augusto Casotti

**Affiliations:** I MSc. Professor, Medicine Course, Universidade Estadual do Tocantins (UNITINS), Augustinópolis (TO), Brazil.; II PhD. Professor, Physical Education Course, Graduate Program in Teaching, Language and Society, Universidade do Estado da Bahia (UNEB), Guanambi (BA), Brazil.; III MSc. Technical Consultant, Health Sciences, Department of Monitoring, Evaluation and Dissemination of Strategic Health Information (DEMAS), Secretariat of Information and Digital Health, Ministry of Health (MS), Brasília (DF), Brazil.; IV MSc. PhD Student, Postgraduate Program in Health Sciences, Universidade Federal de Goiás (UFG), Goiânia (GO), Brazil.; V BS. Physical Education Professional, Universidade Estadual do Sudoeste da Bahia (UESB), Jequié (BA), Brazil.; VI PhD. Professor, Dentistry Course, Graduate Program in Nursing and Health, Universidade Estadual do Sudoeste da Bahia (UESB), Jequié (BA), Brazil.

**Keywords:** Motor activity, Aging, Epidemiology, Sedentary behavior, Muscle strength, Aged, Sitting time, Grip strength, Physical activities, Sedentary time, Elderly

## Abstract

**BACKGROUND:**

Dynapenia is a risk factor of mortality. Therefore, the development of low-cost and easy-to-apply tools is essential to optimize the health surveillance actions of older people.

**OBJECTIVES:**

To compare the time spent on habitual physical activity (HPA) and sedentary behavior (SB) among dynapenic and non-dynapenic older adults and ascertain the predictive ability of these behaviors on outcome.

**DESIGN AND SETTING:**

A cross-sectional population epidemiological survey was conducted involving 208 older adults.

**METHODS:**

HPA and SB were quantified using the International Physical Activity Questionnaire, and dynapenia was identified by handgrip strength (women: 18.37 kgf; men: 26.75 kgf).

**RESULTS:**

The prevalence was 24.50%. In both sexes, dynapenic individuals reported a HPA median time of 70.00 minutes/week (min/wk), while non-dynapenic women and men reported HPA median times of 240.00 and 280.00 min/wk, respectively (P < 0.05). For SB among dynapenic individuals, a median of 388.75 min/day was observed in women and 428.57 min/d in men. In contrast, non-dynapenic women and men had 291.42 and 274.28 min/day in SB (P < 0.05), respectively. The best cutoff HPA to discriminate the outcome was 150.00 min/wk in women (sensitivity: 73.30%; specificity: 60.67%) and 140.00 min/wk in men (sensitivity, 71.43%; specificity, 61.54%). The best cutoff SB was 381.43 min/day in women (sensitivity, 53.30%; specificity, 84.80%) and 351.43 min/day in men (sensitivity, 71.43%; specificity, 73.85%).

**CONCLUSION:**

Older individuals with dynapenia spent less time on HPA and more time in SB. Furthermore, HPA was found to be a better discriminator of dynapenic individuals, and SB better discriminated non-dynapenic individuals.

## INTRODUCTION

Aging is characterized by physiological changes that result in decreased physical fitness and progressive deterioration of musculoskeletal functions.^1,2^ Examples of such changes include decline in muscle strength, which decreases significantly, after 50–60 years of age at a rate of 2.00%–4.00% per year.^3^ This age-related condition is termed dynapenia.^4,5^

The prevalence of dynapenia seems to vary mainly according to the characteristics of each population. For instance, values of 17.80% are found in European older adults,^6^ and 25.10% in Korean older adults.^7^ In Canada, this prevalence ranges from 24.00% to 21.50% in older men and women, respectively.^8^ In the Brazilian older adults population, prevalence of dynapenia is estimated to be approximately 16.60%–25.80% in older men and 17.70%–34.40% in older women.^10^

This situation leads to an adverse epidemiological scenario, considering the implications provided by dynapenia, for the health of older adults, such as greater probabilities of falls,^11^ fractures,^12^ institutionalization and hospitalization.^13^ This outcome is also an important component of frailty syndrome and sarcopenia. Furthermore, older adults with dynapenia are at greater risks of functional disability^14^ and mortality.^15,16^

Handgrip strength (HGS) is a primary criterion for diagnosing muscle weakness. This is because this method is considered the gold standard, as it is a direct measure of functional performance and has a good correlation with global muscle strength.^14^ However, the handheld hydraulic dynamometer, the instrument necessary for such measurement, is unavailable in most healthcare units and/or outpatient clinics, necessitating the requirement of investigating measures that are more accessible to screen this outcome in older adults.^17,18^

There is robust evidence about the connection of the strong impact of habitual physical activity (HPA) and sedentary behavior (SB) on the level of muscle fitness, throughout aging, such that older adults with insufficient levels of physical activity and high exposure to SB demonstrate less strength.^11,19,20^ Therefore, the hypothesis is verified that time spent in HPA, considering different dimensions, such as activities, work, commuting and leisure and the time of sedentary behavior, quantified by validated instruments, such as the International Physical Activity Questionnaire (IPAQ)^21,23^ are presented as a possible strategy to be used for screening muscle weakness in older adults.

However, a literature review shows that no population studies have verified differences in the time spent on HPA and exposure to SB between dynapenic and non-dynapenic older individuals. In addition, studies investigating the accuracy of these risk behaviors in screening the outcome in question are lacking. Therefore, health surveys should be carried out with these perspectives, considering the possibilities of using HPA and SB in the health surveillance framework as a low-cost and easily applied epidemiological tool to screen older adults with a higher probability of low muscular strength.

## OBJECTIVE

To compare the time spent in habitual HPA and SB among dynapenic and non-dynapenic older adults and ascertain the predictive ability of these behaviors on the outcome.

## METHODS

### Study design, location, and population

This was a cross-sectional study, part of the baseline of the population epidemiological survey titled *Health conditions and lifestyle of older individuals from a small municipality: Aiquara cohort.*”^24^ The survey was conducted from February to April 2013 and involved older adults living in the urban areas of Aiquara, Bahia, Brazil and registered in the Family Health Strategy (FHS), which covers 100% of the municipality’s population.^24^ More information about the steps of data collection and procedures adopted can be found in Alves et al.’s study.^25^

Initially, all older adults residing in the urban area of Aiquara, Bahia, were identified through a census conducted in partnership with health professionals working at the FHS. Therefore, households were separated according to the area covered by visiting community health agents. For participation in the health survey, the following inclusion criteria were adopted: age ≥ 60 years; not institutionalized; and having a fixed residence in the urban area (dwelling four days or more at home).

**Figure 1.**
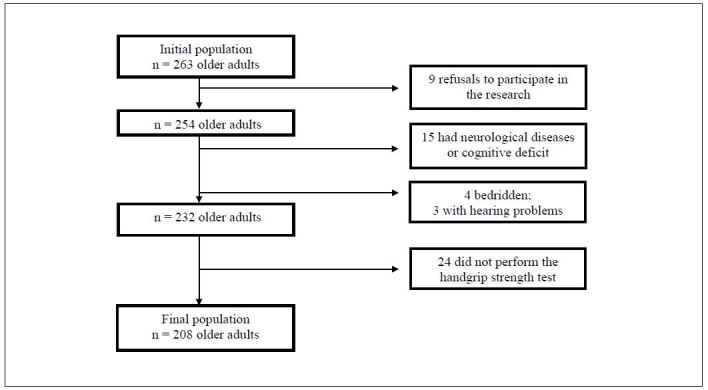
Flow chart depicting the process for including older adults in this study.

Thus, 263 older adults were included in this study. Of these, nine refused to participate in the investigation; 15 were excluded for having a cognitive deficit, verified through the reduced and validated version of the Mini Mental State Examination (MMSE)^26^ with a cutoff < 13^27^ or neurological disease that would hamper the understanding of questions in the interviews, three for having hearing problems, and four for being bedridden. Finally, 232 older adults were selected for the investigation because they met the eligibility criteria.^25^ However, 24 patients were excluded because they did not undergo HGS measurements (Figure1).

### Ethical aspects

All participants were informed of the procedures, and informed consent was obtained before data collection. This survey was conducted in accordance with the ethical principles of the Declaration of Helsinki of the World Medical Association and Resolution No. 466/2012 of the Brazilian National Health Council. This study was approved by the Research Ethics Committee of State University of Southwest Bahia, under Opinion No. 171.464/2012.

### Described variables (population characteristics)

To characterize the population, the following variables were listed: sex (male or female); age (in years); height (Ht), and body mass (BM). Anthropometric parameters were measured as described by Santos et al.^28^ Total muscle mass (TMM) was also considered, estimated using the equation proposed by Lee et al.^29^ and validated for the Brazilian older adults by Rech et al.:^30^ TMM (kg) = (0.244 × BM [kg]) + (7.8 × Ht [m]) – (0.098 × age [years]) + (6.6 × sex) + (ethnicity – 3.3). For the equation constant values, the following values were considered: 1 = male and 0 = female. In addition, ethnicity was self-reported and categorized as follows: 0 = white (white, mestizo, and indigenous); 1.2 = Asian and 1.4 = afro-descendant (black and brown).

### Dependent variable (dynapenia)

The HGS was measured using a Saehan handheld SH5002 hydraulic dynamometer (Saehan Corporation, 973, Yangdeok-Dong, Masan Hoewon-Gu, Changwon 630-728, South Korea). This test involves the upper limb that the participants reported feeling stronger. During the test, the participants remained seated with the arm close to the body, the elbow flexed at 90º, and the forearm in a neutral position. The dynamometer was adjusted according to the size of the individual’s hand such that the first and second joints of the fingers were in flexion.^31^

Throughout the HGS measurement, the participants were encouraged to press the dynamometer handle with as much force as possible. The test was performed twice, with an interval of 1 minute, and the highest value in kgf was used for the analyses. Dynapenia was identified according to sex, with the cutoff point set at the 25^th^ percentile of the HGS (women: ≤ 18.37 kgf; men: ≤ 26.75 kgf).^28^

### Independent variables (habitual physical activity and sedentary behavior)

HPA was verified using the first four domains of the long version of the International Physical Activity Questionnaire,^21^ an instrument validated for older Brazilian adults.^22,23^ Thus, a score was constructed based on the quantification of time spent on moderate-to-vigorous physical activity at work, personal transportation, domestic activities, and leisure.

SB was assessed from the fifth IPAQ domain, which considers the time spent in a sitting or reclining position on weekdays and weekends. Therefore, the weighted average SB was calculated using the following mathematical procedure: (5 × sitting time on a weekday) + (2 × sitting time on a weekend day)/7.^20^

### Statistical analysis

Absolute and relative frequencies, means, medians, standard deviations, interquartile ranges, and percentile values (P25; P75) were calculated to describe the population. In the inferential analyses, the normality distribution of the quantitative variables was initially tested using the Kolmogorov–Smirnov test. For comparisons, Student’s *t*-test was used for normally distributed data (women: Ht; men: Ht, BM, TMM), and the Mann–Whitney U test was used for non-normally distributed data (women: BM, TMM, HPA, SB; men: HPA; SB).

The predictive ability of time spent in the HPA and SB on outcome was verified using the parameters provided by the receiver operating characteristic (ROC) curve. Initially, the accuracy values of each risk behavior were reviewed based on a comparison of the areas under the ROC curve.^32^ Subsequently, the best cutoff points and their relevant sensitivity and specificity values were identified using the Youden index.^33^

In all analyses, a 95.00% confidence interval (CI) and a 5.00% significance level (α ≤ 0.05) were adopted. Statistical procedures were performed using the software IBM SPSS Statistics (version 21.0, 2013, Inc., Chicago, Illinois, United States) and MedCalc (version 19.4.1, 2018, MedCalc Inc., Mariakerke, Belgium).

## RESULTS

This was an epidemiological investigation conducted with 122 women (age, 71.05 ± 6.75 years) and 86 men (age, 72.34 ± 8.17 years). The prevalence of dynapenia was approximately 24.50% (women: 24.60%; men: 24.40%).

Furthermore, it was verified that, in both sexes, dynapenic older adults were shorter and had lower body mass and total muscle mass in comparison with non-dynapenic individuals (P < 0.05; Table 1).

**Table 1.** Comparative analysis of anthropometric parameters of older adults, including women and men and dynapenic and non-dynapenic individuals

WOMEN			
Variable	Non-dynapenic (n = 92)	Dynapenic (n = 30)	P value
Ht (m)*	1.51 (0.61)	1.46 (0.62)	**< 0.001**
BM (kg)_**_	59.45 (19.1)	52.45 (15.3)	**0.017**
TMM (kg)**	17.06 (6.17)	15.49 (4.60)	**0.002**

MEN			
Variable	Non-dynapenic (n = 65)	Dynapenic (n = 21)	P value
Ht (m)*	1.63 (0.61)	1.60 (0.52)	**0.013**
BM (kg)*	67.89 (9.34)	57.79 (9.66)	**< 0.001**
TMM (kg)*	26.99 (2.70)	23.66 (2.90)	**< 0.001**

Ht = height; BM = body mass; TMM = total muscle mass; n = number of individuals per group; *means and standard deviations; **median and interquartile range.

For both women and men, non-dynapenic participants reported spending a longer time on HPA during the week and a shorter time of exposure to SB during the day than dynapenic participants (P < 0.05; Table 2).

**Table 2.** Comparative analysis of habitual physical activity of older adults, including women and men and dynapenic and non-dynapenic individuals

Variables	WOMEN		P value
	Dynapenia		
	No (n = 92)	Yes (n = 30)	
HPA (minutes/week)	240.00*	70.00*	**0.003**
	(P25: 82.50; P75:465.00)	(P25: 22.50; P75: 240.00)	
SB (minutes/day)	291.42*	388.75*	**0.002**
	(P25: 235.75; P75:356.78)	(P25: 262.50; P75: 507.85)	

Variables	MEN		P value
	Dynapenia		
	No (n = 65)	Yes (n = 21)	
HPA (minutes/week)	280.00*	70.00*	**0.015**
	(P25: 95.00; P75:630.00)	(P25: 30.00; P75: 252.50)	
SB (minutes/day)	274.28*	428.57*	**< 0.001**
	(P25: 180.00; P75:320.00)	(P25: 310.00; P75: 360.00)	

HPA = habitual physical activity; SB = sedentary behavior; P = percentile; n = number of individuals per group; *median.

**Figure 2.**
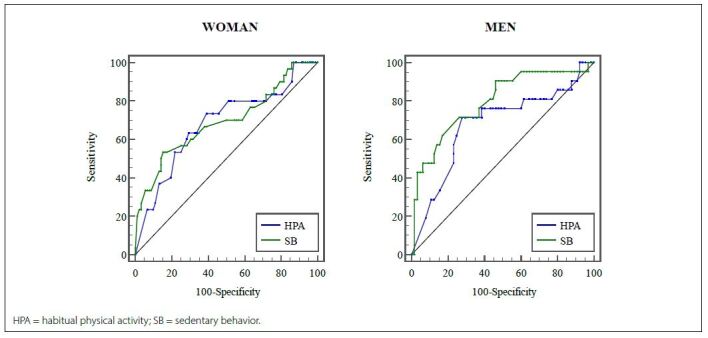
Receiver operating characteristic curves of weekly time in habitual physical activity and daily sedentary behavior for predicting dynapenia in older adults, including women and men

Figure 2 shows the areas under the ROC curve of the weekly time spent on the HPA and the daily time spent on the SB, which were used as discriminators in the dynapenic study population. The variables reviewed presented a lower limit of the CI of the area under the ROC curve > 0.50, with no difference between the accuracies for the two sexes (P > 0.05).

Regarding the other parameters assessed, based on the ROC curve, for women, the best cutoffs for predicting the outcome, based on the weekly time spent in HPA, were 150 min/week (sensitivity: 73.33%; specificity: 60.87%) and 381.43 minutes/day (sensitivity: 53.30%; specificity: 84.80%) for SB. As for men, the best cutoff HPA was approximately 140.00 min/week (sensitivity: 71.43%; specificity: 61.54%) and 351.43 min/d (sensitivity: 71.43%; specificity: 73.85%) for SB (Table 3).

**Table 3.** Receiver operating characteristic curve parameters for weekly time spent in habitual physical activity and daily sedentary behavior, used as predictors of dynapenia in older adults, including women and men

WOMEN				
Variable	Cutoff point	Sensitivity (%)	Specificity (%)	AUC (95%CI)
HPA (minutes/week)	150.00	73.33 (54.10–87.70)	60.87 (50.10–70.90)	0.68 (0.59–0.76)
SB (minutes/day)	381.43	53.30 (44.30–71.70)	84.80 (75.80–91.40)	0.69 (0.60–0.77)

MEN				
Variable	Cutoff point	Sensitivity (95%CI)	Specificity (95%CI)	AUC (95%CI)
HPA (minutes/week)	140.00	71.43 (47.80–88.70)	61.54 (48.60–73.30)	0.68 (0.55–0.79)
SB (minutes/day)	351.43	71.43 (47.80–88.70)	73.85 (61.50–84.00)	0.79 (0.69–0.87)

HPA = habitual physical activity; SB = sedentary behavior; AUC = area under the receiver operating characteristic curve; CI = confidence interval. HPA = habitual physical activity; SB = sedentary behavior.

## DISCUSSION

This was the first investigation into the predictive capacity of the time spent weekly with the HPA and the time spent daily with the SB for screening dynapenia in older adults. The main results were that, in both sexes, dynapenic participants spent a shorter time in the HPA and had greater exposure to SB. The two risk behaviors reviewed showed accuracy ≥ 68.00%, with no significant difference between them, with a lower limit of the CI > 50.00%, which warrant them as potential indicators for discrimination of the outcome.

A systematic review of 112 epidemiological surveys was carried out, totaling 43,796 community-dwelling older adults. Longer time in HPA and lower exposure to SB were associated with higher muscle strength.^19^ Congruently, a previous study carried out by our research group, with the older adult population of Aiquara, Bahia, showed that those with weekly time < 150 minutes in HPA and high exposure to SB, identified with the cutoff point set at the 75^th^ percentile of sitting/leaning backwards time (≥ 382.85 minutes/day), had greater probabilities to develop dynapenia, with prevalences ratios referred to 1.99 (95% CI: 1.12–3.54) and 1.88 (95% CI: 1.19–2.98), respectively.^20^

The apparent positive relationship between HPA and muscle strength and the negative relationship between SB and muscle strength in older adults are probably a consequence of harmful physiological repercussions on health arising from hypokinetics. This is because low motor activity generates adverse effects in different systems of the human body, such as the muscles: atrophy and decrease in the number of muscle fibers, particularly the IIA and IIX; reduction of genes involved in mitochondrial function and volume and oxidative capacity; and decline in the capacity of key proteins responsible for glucose transport and storage within the skeletal muscles, which impairs the process of phosphorylation to obtain energy for muscle contractions.^34^

Furthermore, the disuse of the skeletal muscles, caused by the short time spent in HPA and by the high exposure to SB, can cause excessive accumulation of body fat and its infiltration into the muscle cells, weakening their stimulation and contraction power.^35^ Together such repercussions lead to the declines in the ability of muscle groups to maintain a dynamic balance and yield strength and power, culminating in muscle weakness.^34^

Nevertheless, the findings of the present study showed that, in both sexes, the HPA was more sensitive to dynapenia, with cutoff points close to those suggested by the World Health Organization for a sufficient level of physical activity (150 minutes/week).^36^ However, the SB proved to be more specific, with cutoff points > 350 minutes/day for men and > 381.43 min/d for women, which is closer, particularly for women, to the value corresponding to the 75^th^ percentile of the SB-weighted average, verified in the older population of Aiquara-BA by Santos et al.^20^

These findings suggest that in the absence of a dynamometer in the framework of healthcare, HPA appears to be an epidemiological indicator to initially track older adults who are more likely to experience dynapenia, that is, those that are truly positive. Subsequently, the SB, as it demonstrated greater specificity, can be adopted as an indicator to track those older adults who really do not have dynapenia, i.e., true negatives. This complements screening for dynapenia in community-dwelling older adults.

Another relevant verification observed among older adults from Aiquara, Bahia, concerns a smaller contingent of the total muscle mass in dynapenic participants of both sexes, compared to that in non-dynapenic individuals. This observation points to the possibility that older adults with dynapenia are more prone to developing sarcopenia, a chronic muscle disease characterized by muscle weakness concomitant with low muscle mass, according to the criteria of the European Working Group on Sarcopenia in Older People.^14^

We found in the literature that an active lifestyle consisting mainly of counter-resistance exercises a main strategy to improve or maintain strength and muscle mass throughout aging. Therefore, the National Strength and Conditioning Association suggests for seniors 2–3 days of resistance training, consisting of 1–2 exercises for major muscle groups (8–10 total exercises) at progressive intensity, until reaching 70.00%–85.00% of the maximum recommended repetition of each exercise throughout the periodization.^37^

With regards to mitigating the noxious effect of SB on muscle fitness, the implementation of breaks in low-energy expenditure activities is a possibility to be considered.^38^ This is because the simple act of leaving the sitting or leaning position to get up and move around, for example, or simply stretching the body, results in the need to recruit the skeletal muscles to maintain the orthostatic position against gravity, raising the energy expenditure above the resting level.^39^

This investigation had some limitations, such as the quantification of muscle mass in older adults using an anthropometric equation, which, despite being validated, is not considered the gold standard. Another limitation lies in the reliance on self-reporting of HPA and SB measurements, as they are prone to recall bias, which may result in overestimation or underestimation of the actual time spent on the activities in question. However, the prior training of the survey team concerning the standardization of information acquisition is worth mentioning. Furthermore, in the present study, MMSE was used as a way to identify those older adults with cognitive deficits, with the aim of reducing the impact of their memory bias.

This study also had strengths, such as: the hand hydraulic dynamometry used to measure muscle strength, as it is considered a gold-standard method for identifying dynapenia. In addition, the census perspective should be highlighted, which allowed evaluation of the older adult population of a small municipality in the Brazilian Northeast, which has low values in socioeconomic/demographic indicators and, therefore, presents limitations in provision of health services.^24^

Thus, the proposition of using the weekly time spent on HPA and daily SB presents itself as an epidemiological, financially accessible, and easy-to-apply strategy that can be used by any health professional familiar with the IPAQ and active in primary healthcare, not only in Aiquara, Bahia, but also in other municipalities with similar characteristics, to screen older adults with a higher probability of dynapenia.

Finally, the low sensitivity of SB for screening dynapenia in women may be a consequence of disparities in this behavioral pattern between older women and men. Another hypothesis concerns the possibility that it is easier to quantify the time spent on this risky behavior in older men than in older women, which in turn raises the requirement of future studies that aim to provide evidence for this issue.

## CONCLUSIONS

This evidence supports our hypothesis. In the present study, among older adults of both sexes, the dynapenic participants spent a shorter time in the HPA and a longer time in the SB. In addition, the HPA showed a better ability to discriminate dynapenic older adults, and the SB was a better discriminator for non-dynapenic individuals. These findings suggest the possibility of using these two indicators concomitantly for better screening of dynapenia in community-dwelling older adults in the absence of a hydraulic dynamometer to measure HGS.
